# Mantodea, Blattodea, Orthoptera, Dermaptera, and Phasmida of Canada

**DOI:** 10.3897/zookeys.819.27241

**Published:** 2019-01-24

**Authors:** James Miskelly, Steven M. Paiero

**Affiliations:** 1 Royal British Columbia Museum, 675 Belleville St., Victoria, British Columbia, V8W 9W2, Canada Royal British Columbia Museum Victoria Canada; 2 School of Environmental Sciences, 50 Stone Rd. East, University of Guelph, Guelph, Ontario, N1G 2W1, Canada University of Guelph Guelph Canada

**Keywords:** biodiversity assessment, Biota of Canada, Blattodea, cockroaches, crickets, Dermaptera, earwigs, grasshoppers, katydids, mantids, Mantodea, Orthoptera, Phasmida, stick insects, termites

## Abstract

In the last 40 years, the number of species in the orthopteroid orders has increased by ~10% from that known in 1979. The largest order, the Orthoptera, has increased from 205 to 235 species known in Canada. The number of Blattodea has increased from 14 to 18 species, while Dermaptera has increased from 5 to 6 species. The number of species of Mantodea (3) and Phasmida (1) known in Canada have remained unchanged. Most new species records reported in Canada since 1979 have resulted from new collections along the periphery of the range of more widespread species. Some species reported since 1979 are recent introductions to Canada, including species restricted to homes or other heated buildings. The taxonomy of these orders has also changed, with only the Dermaptera having maintained its order definition since the 1979 treatment. Additional orthopteroid species are likely to occur in Canada, particularly in the orders Orthoptera and Blattodea. DNA barcodes are available for more than 60% of the species known to occur in Canada.

## Introduction

The insects treated here represent part of the cohort Polyneoptera, a group that also includes the orders Embioptera, Grylloblattodea, Mantophasmatodea, Plecoptera, and Zoraptera (Song et al. 2016), with the Canadian representatives of Plecoptera and Grylloblattodea being treated elsewhere in this series. The orders of the Polyneoptera dealt with here are informally referred to as the “orthopteroids”, based on the obsolete and paraphyletic superorder Orthopteroidea (Eades 2018), but for the convenience of dealing with this paraphyletic assemblage we continue to use it here. Since 1979, the order-level taxonomy has changed for most of the orthopteroids. The Mantodea (mantids) was reviewed as a part of the Dictyoptera (spelled Dictuoptera in the original) by Kevan (1979b) but is now treated as a separate order (Zhang 2013, Otte et al. 2018, Wieland and Svenson 2018). Kevan (1979b) also included the Blattodea (cockroaches) and Isoptera (termites) within the Dictyoptera, but they are now treated together as the Blattodea (Inward et al. 2007, Djernæs et al. 2012). Kevan (1979c, d) presented saltatorial orthopteroids as two orders, Orthoptera s. str. and Grylloptera. The current taxonomic system recognizes both of these within the order Orthoptera, and treats Kevan’s divisions as the suborders Caelifera (grasshoppers) and Ensifera (crickets and allies), respectively (Cigliano et al. 2018). The order Phasmida (stick insects) was reviewed under the name Cheleutoptera by Kevan (1979a) but now follows Brock et al. (2018), while the Dermaptera (earwigs) has remained unchanged.

Shortly after the 1979 reviews (Kevan 1979a, b, c, d, Lamb 1979), an authoritative information source was published, covering all of the Polyneoptera of Canada except Plecoptera (Vickery and Kevan 1985). This manual brought together for the first time all of the known ecological and distributional information available, and keys to all Canadian species. It also provided species lists that were based on largely the same information as the 1979 reviews and, due to the short period of time between the two publications, serve as a good proxy for the information available at the time of the 1979 reviews for comparison with the current examination. The work by Vickery and Kevan (1985) provided the foundation for virtually all subsequent study of orthopteroid orders in Canada. Advances in taxonomy and discoveries of new species have resulted in some current limitations to the 1985 manual, but there remains no single reference that can replace it.

Research in recent decades has benefitted from tools not available to earlier authors. Genetic evidence has revealed cryptic species (e.g., Guillet et al. 2000) and clarified higher taxonomic relationships (e.g., Djernæs et al. 2012). The proliferation of DNA barcoding and the development of the Barcode Index Number (BIN) system have proved useful in the recognition of new species (Hebert et al. 2003, Ratnasingham and Hebert 2007, 2013), and further exploration of the BIN data may improve the understanding of Canadian orthopteroids.

Most of the orthopteroid orders are relatively species-poor and well-sampled in Canada. As a result, our understanding of the diversity and distribution of these orders in Canada has changed little since the 1979 review, with the most substantial species increases occurring in the Orthoptera. The orthopteroid orders are much more diverse in warmer climates with an estimated 40,000 species worldwide (Zhang 2013). The highest diversity in Canada is in the southern parts of British Columbia (BC), the Prairie Provinces, and Ontario (ON). Despite southern BC and ON being the most intensively sampled areas, they remain the areas where most new species are found and where most potential additional species are likely to be found. Additionally, due to their low diversity, relatively large body size, and ease of identification, there is a lot of active interest in the orthopteroids within both the scientific and naturalist communities, especially in the Orthoptera due to their acoustic abilities and behaviours. As a result, new ecological and distributional data are being documented not just in the scientific literature, but also in photograph-sharing fora such as BugGuide.net and iNaturalist, where citizen scientists are contributing to our understanding of these orders.

## Mantodea

There has been no change to the number of mantid species known in Canada since Kevan’s (1979b) review, with one native and two introduced species recorded (Table 1). The European mantis, *Mantisreligiosa* (Linnaeus), was listed by Kevan (1979b) as occurring only in ON and Quebec (QC), but was subsequently mapped by Vickery and Kevan (1985) as also occurring in a small area of BC. This mantid has since expanded its range dramatically in southern BC (Cannings 2007). The native ground mantid, *Litaneutriaminor* Scudder, was listed as occurring in both BC and Alberta by Kevan (1979b) and Vickery and Scudder (1987); however, some publications have stated that the species’ Canadian range is restricted to BC only (Vickery and Kevan 1985, Cannings and Cannings 1995). A subsequent record from Saskatchewan (Hooper 2003) was an incorrectly identified mantispid (C Sheffield pers. comm.). *Tenoderasinensis* Saussure, is an Asian species established in ON and QC. One additional species, *Tenoderaangustipennis* Saussure, an Asian species established as far north as New York (Gurney 1951), may eventually be found in Canada.

The Mantodea of Canada represent 15% of the 20 species known from North America north of Mexico (Iowa State University 2003–2018). BINs are available for both of the introduced mantids in Canada, but the native ground mantid has not been barcoded to date (BOLD Systems 2018) (Table 1).

In total, the Canadian Blattodea consists of 18 breeding species, including 12 cockroaches and six termites (Table 2). This is an increase of five breeding species (three cockroaches and two termites) from Kevan (1979b). *Periplanetabrunnea* Burmeister was apparently treated as established in Kevan (1979b) but actually did not successfully establish in Canada (Vickery and Kevan 1985). The Canadian established cockroach fauna represents ~18% of the 66 North American cockroach species based on Pratt (1988) plus recently introduced species, and the termite fauna represents ~13% of the 46 North American species based on Constantino (1998). Only four cockroach species and five termite species recorded in Canada are native, with the remainder introduced. Additional species may become established in Canada, including several of the cultured cockroach species (Pratt 1988) that could potentially become established in human dwellings. Two additional species of *Ectobius* introduced to the northeastern United States (Hoebeke and Nickle 1981, Hoebeke and Carter 2010) may also occur in eastern Canada. One new termite species in the family Archotermopsidae has been documented in BC and the western United States (Szalanski et al. 2006) based on genetic profiles but currently remains undescribed. All 12 Canadian cockroach species and six termite species have corresponding BINs (Table 2).

**Table 1.** Census of Mantodea in Canada.

**Taxon^1^**	**No. species reported by Kevan (1979b)**	**No. species currently known in Canada^2^**	**No. BINs^3^ available for Canadian species**	**Est. no. undescribed or unrecorded species in Canada**	**General distribution by ecozone^4^**	**Information sources**
Mantidae	3	3 (2)	2	1	Pacific Maritime, Montane Cordillera, Western Interior Basin, Prairies, Mixedwood Plains, Hudson Plains, Atlantic Maritime	Vickery and Kevan 1985, Cannings 2007

^1^Classification follows Otte et al. (2018). ^2^The number in parentheses represents the number of established non-native species included in the total. ^3^Barcode Index Number, as defined in Ratnasingham and Hebert (2013). ^4^See figure 1 in Langor (2019) for a map of ecozones.

## Blattodea

The Blattodea fauna of Canada, including the cockroaches and termites, is well known. Although largely perceived as pests due to a number of non-native species that either occur in human dwellings or damage lumber, Canada has several native species that occur in and around forest environments. This native element of the Canadian fauna has remained relatively unchanged since Kevan (1979b), while the introduced fauna has grown due to the establishment of recently introduced species to North America. Vickery and Kevan (1985) provide keys, natural history information, and distributional data for most of the Canadian cockroach and termite species, and Hoebeke and Carter (2010) provide keys to the *Ectobius* cockroach species that have become established in North America. Important resources on general cockroach biology, distributional data and natural history include Bell et al. (2007) and Cockroach Species File Online (Beccaloni 2014). Important resources for termite biology and identification of North America termites include Weesner (1965), Constantino (1998), Bignell et al. (2011), Evans et al. (2013) and Krishna et al. (2013).

There are also a number of species that occur in Canada but are not considered established outside of cultures. A number of cockroach species (~10–20 species) are actively cultured as pets or as a food source in the pet trade for reptiles and amphibians in North America, and some may escape these cultures to become established for short periods in buildings. Cockroaches are also regularly intercepted from international shipments into Canada; Vickery and Kevan (1985) provided a list of 18 cockroach species that have been intercepted in Canada but are not known to be established. Among the termites, several termite species recognized as pests to lumber products in the USA have occasionally been transported into Canada (Vickery and Kevan 1985, Grace et al. 1991) but have never successfully established. All of these species are excluded from Table 2.

**Table 2.** Census of Blattodea in Canada.

Taxon^1^	No. species reported in Kevan (1979b)	No. species currently known in Canada^2^	No. BINs^3^ available for Canadian species	Est. no. undescribed or unrecorded species in Canada	General distribution by ecozone^4^	Information sources
**Superfamily Blaberoidea**						
Blaberidae	1	1 (1)	0	0	domiciliary; Mixedwood Plains, Pacific Maritimes, likely in other southern ecozones	Vickery and Kevan 1985, Cannings and Scudder 2005a, Paiero and Marshall 2014
Ectobiidae	5	8 (4)	7	4–5	Hudson Plains; domiciliary in all southern ecozones	Vickery and Kevan 1985, Chandler 1992, Cannings and Scudder 2005a, Hoebeke and Carter 2010, Klassen and Sharanowski 2014, Paiero and Marshall 2014, Clements et al. 2017
**Superfamily Blattoidea**						
**Epifamily Blattoidae**						
Blattidae	4	3 (3)	2	1–2	domiciliary in all southern ecozones	Kevan 1979b, Vickery and Kevan 1985, Cannings and Scudder 2005a, Paiero and Marshall 2014
**Epifamily Termitoidae**						
Archotermopsidae	2	2	1	0	Pacific Maritime, Montane Cordillera, Western Interior Basin	Vickery and Kevan 1985, Cannings and Scudder 2005c
Rhinotermitidae	2	4 (1)	3	1	Pacific Maritime, Mixedwood Plains	Vickery and Kevan 1985, Cannings and Scudder 2005c, Szalanski et al. 2006
**Total**	**14**	**18 (9)**	**13**	**6–8**		

^1^Classification follows that indicated in Beccaloni 2014 and Engel 2011. ^2^The number in parentheses represents the number of established non-native species included in the total. ^3^Barcode Index Number, as defined in Ratnasingham and Hebert (2013). ^4^See figure 1 in Langor (2019) for a map of ecozones.

## Orthoptera

Much more is known about the Orthoptera (grasshoppers, locusts, crickets, katydids) of Canada today than when Kevan reviewed this group in 1979 (Kevan 1979c, d). New field work has improved our understanding of species’ distributions within Canada: in the north (Vickery 1997, Catling 2008), BC (Miskelly 2012), the Prairie Provinces (Johnson 2001, 2002, 2003), ON (Marshall et al. 2004, Paiero and Marshall 2014), and Atlantic Canada (McAlpine and Ogden 2012, Catling et al. 2013, McAlpine et al. 2015). Other studies have focussed on the biogeographical affinities and ecological associations of this group (Vickery 1986, Scudder and Vickery 2011, Miskelly 2014). A great deal of research has also been done in the fields of behaviour and acoustics (e.g., Gwynne 1977, 1982, Tuckerman et al. 1993, Morris et al. 2002, Judge 2011).

The 1979 assessment listed the known Orthoptera of Canada as 217 species in eleven currently recognized families (Table 3), with an estimated 24 additional unreported or undescribed species expected. However, because the original review did not present an actual species list, it is difficult to interpret potential changes in taxonomy or species identifications since 1979. Luckily, there were few known changes between the 1979 review and the publication of Vickery and Kevan (1985). The species lists contained in that reference are presumed to be very similar to the information that formed the basis of the 1979 review. Based on the species lists available in Vickery and Kevan (1985), it appears that the species number used in the 1979 may have been inflated by the inclusion of subspecies. Since the publication of the Vickery and Kevan (1985), a lot of changes have been made in the recognition of subspecies. Many subspecies that likely contributed to species counts in Vickery and Kevan (1985), and likely also in Kevan (1979c, d) are no longer recognized, while some others have been raised to species status. In addition, several species believed by Vickery and Kevan to occur in Canada were based on misidentifications (Miskelly 2012). A review of Vickery and Kevan (1985) shows that only 205 currently-recognized species were known to occur in Canada at the time of the original review, which would reduce the species recognized in 1979 by 14. In 2018, the known Orthoptera of Canada total 235 species in 12 families (Table 3). This number represents 19% of the roughly 1200 species known to occur in North America north of Mexico (Iowa State University 2003–2018).

**Table 3.** Census of Orthoptera in Canada.

Taxon^1^	No. species reported in Kevan (1979c, d) ^2^	No. species currently known in Canada^3^	No. BINs^4^ available for Canadian species	Est. no. undescribed or unrecorded species in Canada	General distribution by ecozones^5^	Information sources
**Suborder Caelifera**						
Acrididae	117	129 (1)	83	4	all ecozones	Vickery and Scudder 1987, Miskelly 2012, Paiero and Marshall 2014
Tetrigidae	7	7	7	0	all ecozones except Arctic	Vickery and Scudder 1987, Miskelly 2012, Paiero and Marshall 2014
Tridactylidae	2	3	0	0	Mixedwood Plains	Vickery and Scudder 1987, Paiero and Marshall 2014
**Suborder Ensifera**						
Gryllidae	11	16 (2)	11	2	all ecozones south of taiga	Vickery and Scudder 1987, Miskelly 2012, Paiero and Marshall 2014
Trigonidiidae	7	9 (2)	8	1	all ecozones south of taiga	Vickery and Scudder 1987, Miskelly 2012, Paiero and Marshall 2014
Gryllotalpidae	1	1	0	0	Mixedwood Plains	Paiero and Marshall 2014
Myrmecophilidae	1	2	1	1	Pacific Maritime, Western Interior Basin, Mixedwood Plains	Miskelly 2012, Paiero and Marshall 2014
Prophalangopsidae	2	2	4	1	Pacific Maritime, Western Interior Basin, Montane Cordillera	Vickery and Scudder 1987, Miskelly 2012
Gryllacrididae	0	1	0	0	Mixedwood Plains	Paiero and Marshall 2014
Stenopelmatidae	2	2	1	0	Pacific Maritime, Western Interior Basin, Prairie	Vickery and Scudder 1987, Miskelly 2012
Tettigoniidae	36	44 (4)	29	6	all southern ecozones, reaching northern limit in Boreal Plains	Vickery and Scudder 1987, Miskelly 2012, Paiero and Marshall 2014
Rhaphidophoridae	19	20 (1)	13	0	all southern ecozones, reaching northern limit in Boreal Plains	Vickery and Scudder 1987, Miskelly 2012, Paiero and Marshall 2014
**Total**	**205**	**235 (10)**	**157**	**15**		

^1^Taxonomy follows Cigliano et al. (2018). ^2^This number has been reconciled with Vickery and Kevan (1985) to exclude subspecies (see text for further explanation). ^3^The number in parentheses represents the number of established non-native species included in the total. ^4^Barcode Index Number, as defined in Ratnasingham and Hebert (2013). ^5^See figure 1 in Langor (2019) for a map of ecozones.

The number of additional species expected in Canada (Table 3) but which are yet undocumented (undiscovered or undescribed) was estimated by examination of the distribution maps and records contained in Vickery and Kevan (1985), Iowa State University (2003–2018), and Walker (2018). The species considered most likely to eventually be found in Canada are those that occur close to the Canadian border in habitats that also occur in Canada, as well as non-native species that are spreading rapidly in the United States. Several undescribed or unrecognized species are also believed to already exist in Canadian collections. These include recent collections that have not yet been definitively identified, as well as potential cryptic species whose existence is suggested by DNA barcoding results.

DNA barcodes have been generated for the majority of Canadian Orthoptera species, resulting in 157 BINs (Table 3) (BOLD Systems 2018). The most common species in Canadian collections are, of course, the species most represented in the database. For species rarely collected in Canada, such as members of the families Gryllacrididae and Gryllotalpidae, there are no BINs that correspond to Canadian specimens; however, some of these species have BINs corresponding to specimens collected in the United States.

The Orthoptera of Canada are most diverse in the southern parts of BC, ON, and the Prairie Provinces, and these are the areas where the majority of new species added to the Canadian list since 1979 have come from (Vickery and Scudder 1987, Marshall et al. 2004, Miskelly 2012, 2013, Paiero and Marshall 2014). The great majority of Orthoptera species in Canada are native. Of the known introduced species, four are established species that live outdoors, and three are domiciliary species that don’t persist outdoors. Two species are accidental introductions that are known only from butterfly conservatories, where they live in artificially maintained tropical conditions (Paiero and Marshall 2014). Because these species are not cultured or deliberately maintained, they are included in Table 3. Only two Canadian species have Holarctic distributions and three species are endemic to Canada (Vickery and Kevan 1985). One species, *Melanoplusspretus* (Walsh), that was formerly found in Canada and USA is believed to be extinct (Lockwood and Debrey 1990). Dead individuals of this species are known to occur in glaciers in USA, and could occur in similar situations in the Canadian Rocky Mountains (Lockwood et al. 1991).

## Dermaptera

The earwig fauna of Canada has changed very little since 1979. As a relatively small order of relatively large and conspicuous insects, the earwigs of North America are well known and well documented, with several regional keys available, including to species of Canada (Vickery and Kevan 1985) and eastern North America (Hoffman 1987). Engel (2003) also provides a key to the earwig genera of North America, while Hoffman (1987) provides a checklist for North America, but additional adventitious species have certainly been recorded since (e.g., Choate 2001). The biology and behaviour of Canadian earwigs was covered in detail by Vickery and Kevan (1985). New records and distributional information comes from recent regional checklists of ON (Paiero and Marshall 2016) and BC (Cannings and Scudder 2005b) while Guillet et al. (2000) discuss the differences in behaviour of the two *Forficula* species established in Canada.

For Canadian species, Vickery and Kevan (1985) provide keys and distributional data for all of the established species, and the distributions have changed very little. The fauna consists of six breeding species, an increase from the five species that Lamb (1979) recorded (Table 4). Most of the species were inadvertently introduced to Canada. The only change to our breeding fauna is the treatment of the European Earwig (*Forficulaauricularia* Linnaeus) as a pair of cryptic species (Guillet et al. 2000), only separable by genetic or phenotypic characters. Two additional species have been recorded in Canada, *Dorutaeniatum* (Dohrn) and *Maravaarachidis* (Yersin) (Paiero and Marshall 2014), but these were apparently short term indoor establishments. Vickery and Kevan (1985) also include two other species (*Chelidurellaacanthopygia* (Gene) and *Dorudavisi* Rehn and Hebard) as adventitious. *Chelidurellaacanthopygia* was recorded from intercepted goods but it is unclear if *D.davisi* was intercepted or if it was an adventive. None of these four species are included in the species total in Table 4 as they were either intercepted individuals or are no longer established. In total, the Canadian fauna represents ~35% of the 17 North American species based on Hoffman (1987), with 80% of the Canadian fauna (including adventitious species) non-native. There are currently only four BINs available for Canadian earwig species (Table 4), representing 66% of the fauna.

**Table 4.** Census of Dermaptera in Canada.

Taxon^1^	No. species reported in Lamb (1979)^2^	No. species currently known in Canada^3^	No. BINs^4^ available for Canadian species	Est. no. undescribed or unrecorded species in Canada	General distribution by ecozone^5^	Information sources
Anisolabididae	2	2 (1)	1	0	in greenhouses; Pacific Maritime, Mixedwood Plains	Cannings and Scudder 2005b, Paiero and Marshall 2014, Vickery and Kevan 1985
Forficulidae	2	3 (2)	2	0	urban areas and buildings in all ecozones south of taiga	Cannings and Scudder 2005b, Paiero and Marshall 2014, Vickery and Kevan 1985
Sphongiphoridae	1	1 (1)	1	0	urban areas and buildings in all southern ecozones	Cannings and Scudder 2005b, Paiero and Marshall 2014, Vickery and Kevan 1985
**Total**	**5**	**6 (4)**	**4**	**0**		

^1^Classification follows that of Hopkins et al. (2018). ^2^Lamb (1979) did not provide a breakdown by family. ^3^The number in parentheses represents the number of stablished non-native species included in the total. ^4^Barcode Index Number, as defined in Ratnasingham and Hebert (2013). ^5^See figure 1 in Langor (2019) for a map of ecozones.

## Phasmida

There has been no change to the number of phasmid species in Canada or to the known distribution since the original review by Kevan (1979a). There remains a single Canadian species, *Diapheromerafemorata* (Say), that is native to ON, QC, and Manitoba (MB) (Vickery and Kevan 1985) (Table 5). Kevan (1979a) stated that two other species may eventually be found in Canada. *Diapheromeravellii* Walsh is recorded from prairie remnants as far north as Minnesota (Vickery and Kevan 1985) and should be looked for in similar habitat in MB and western ON. *Carausiusmorosus* (Sinety) is native to India, sometimes cultivated in the pet trade, and has become established in California as far north as the San Francisco area (Headrick 2011); however, as this species has not spread into any area that regularly experiences freezing weather in the winter, it is not likely to become established in Canada and is not included in Table 5.

The single species of Phasmida in Canada represents 3% of the 29 species known from North America north of Mexico (Iowa State University 2003–2018). This species is represented in the Barcode of Life Database as a single BIN (BOLD Systems 2018) (Table 5).

**Table 5.** Census of Phasmida in Canada.

**Family^1^**	**No. species Reported by Kevan (1979a)**	**No. species currently known in Canada**	**No. BINs available for Canadian species^2^**	**Est. no. undescribed or unrecorded species in Canada**	**General distribution by ecozone^3^**	**Information sources**
Diapheromeridae	1	1	1	1	Mixedwood Plains, Prairies	Vickery and Kevan 1985

^1^Classification follows Brock et al. (2018). ^2^Barcode Index Number, as defined in Ratnasingham and Hebert (2013). ^3^See figure 1 in Langor (2019) for a map of ecozones.

## Future studies and opportunities

With the expanding interest in orthopteroids, our knowledge of the natural histories of Canadian species will benefit from the various web portals that allow professional and citizen scientists to share information. This includes expanding our understanding of habitat requirements for native species, along with the impact that non-native species, such as the recently established *Ectobius* species, may have on our natural areas. Additional advancements can be made in documenting the full distribution of our native species, especially those that are not easily recognized and rarely encountered. Interest in orthopteroids can also be further enhanced with digital guides to the Canadian fauna.

While the orthopteroid fauna is relatively small and well known in Canada, compared with several of the larger orders, we do expect that there will be additional species, both native and non-native, that will occur here, especially within the Orthoptera. Continued sampling in natural areas along the southern regions of Canada, especially ON and BC, will likely document additional native species whose northern limits extend just across the American border. Further sampling in some provinces will also help fill gaps in our distributional data. Continued sampling of both urban and natural areas may also find additional non-native species and help document the spread of already established species. Finally, while we continue to utilize morphological characters to recognize the majority of species, the utilization of additional behavioural and molecular datasets may help us recognize previously undocumented cryptic species, such as in the Orthoptera (e.g., *Oecanthus*) where several species within a species complex are expected to occur in Canada.
